# Cerebral Organoids for Modeling of HSV-1-Induced-Amyloid β Associated Neuropathology and Phenotypic Rescue

**DOI:** 10.3390/ijms23115981

**Published:** 2022-05-26

**Authors:** Haowen Qiao, Wen Zhao, Moujian Guo, Lili Zhu, Tao Chen, Jibo Wang, Xiaodong Xu, Zhentao Zhang, Ying Wu, Pu Chen

**Affiliations:** 1 Tissue Engineering and Organ Manufacturing (TEOM) Lab, Department of Biomedical Engineering, Wuhan University TaiKang Medical School (School of Basic Medical Sciences), Wuhan 430071, China; haowenqiao@whu.edu.cn (H.Q.); zw1018@whu.edu.cn (W.Z.); lilizhu@whu.edu.cn (L.Z.); taochen1014@whu.edu.cn (T.C.); wangjibo@whu.edu.cn (J.W.); xuxiaodong@whu.edu.cn (X.X.); 2 Hubei Province Key Laboratory of Allergy and Immunology, Wuhan University TaiKang Medical School (School of Basic Medical Sciences), Wuhan 430071, China; mjguo@whu.edu.cn; 3 State Key Laboratory of Virology, Wuhan University, Wuhan 430071, China; 4 Institute of Medical Virology, Wuhan University TaiKang Medical School (School of Basic Medical Sciences), Wuhan 430071, China; 5 Department of Neurology, Renmin Hospital of Wuhan University, Wuhan 430050, China; zhentaozhang@whu.edu.cn

**Keywords:** disease modeling, neurodegeneration, cerebral organoid, AD viral hypothesis, pharmacologic treatments

## Abstract

Herpes simplex virus type I (HSV-1) infection is a potential risk factor involved in the Amyloid β (Aβ) associated neuropathology. However, further understanding of the neuropathological effects of the HSV-1 infection is hampered by the limitations of existing infection models due to the distinct differences between human brains and other mammalians’ brains. Here we generated cerebral organoid models derived from pluripotent stem cells to investigate the HSV-induced Aβ associated neuropathology and the role of antiviral drugs in the phenotypic rescue. Our results identified that the HSV-1-infected cerebral organoids recapitulated Aβ associated neuropathology including the multicellular Aβ deposition, dysregulated endogenous AD mediators, reactive gliosis, neuroinflammation, and neural loss, indicating that cerebral organoids offer an opportunity for modeling the interaction of HSV-1 with the complex phenotypes across the genetic, cellular, and tissue levels of the human Alzheimer’s disease (AD). Furthermore, we identified that two antiviral drugs, namely Ribavirin (RBV) and Valacyclovir (VCV), inhibited HSV-1 replication and rescued the neuropathological phenotypes associated with AD in the HSV-1-infected cerebral organoids, implying their therapeutic potential to slow down the progression of AD. Our study provides a high-fidelity human-relevant in-vitro HSV-1 infection model to reconstitute the multiscale neuropathological features associated with AD and discover therapeutic drug candidates relevant to the AD viral hypothesis.

## 1. Introduction

Alzheimer’s disease (AD) is a progressive neurodegenerative disorder characterized by neuronal death, brain atrophy, cognitive impairment, and ultimately a decline in daily life activities [1]. The cost of AD healthcare is $818 billion in 2015 worldwide and could rise as high as $2 trillion by 2030 [2]. Approximately 50 million individuals around the world suffer from AD, and the number of AD patients is predicted to be 152 million in 2050 according to the World Alzheimer Report [3]. Therefore, there is an urgent need for probing AD pathogenic mechanisms and develops effective therapeutics.

Clinically, the common neuropathological hallmarks of AD are mainly characterized by the extracellular sediment of insoluble aggregates amyloid-β protein (Aβ) in the cortex and subcortex, and the formation of intracellular neurofibrillary tangles by hyperphosphorylated tau proteins. These pathologic features are commonly accompanied by the loss of neurons, neuroinflammation, and reactive gliosis [4]. There exist various hypotheses regarding the AD etiology, including the amyloid cascade hypothesis, tau propagation hypothesis, cholinergic hypothesis, inflammatory hypothesis, viral hypothesis, and so on [5]. Notably, some recent studies have provided intriguing implications that the AD viral hypothesis and the amyloid cascade hypothesis could be complementary with each other and provide an intrinsic and consistent interpretation that the infection can trigger the Aβ production, ultimately resulting in the onset of AD pathology. However, the ultimate etiology of AD remains controversial.

In recent years, there is increasingly accumulating evidence indicating that AD may be associated with various pathogens, such as herpes simplex virus type I (HSV-1) and cytomegalovirus [6]. Especially, HSV-1 is highly prevalent and capable of establishing lifelong infection, which accounts for a heavy disease burden in both life quality and economy [7]. HSV-1 is a human-specific virus that establishes life-long latency mainly in the sensory neurons of trigeminal ganglia. The virus can periodically reactivate and travel along axons to the site of primary infections [8]. The clinical studies [9] have reported a molecular, genetic, and clinical network analysis of virome from the human post-mortem brains and concluded that levels of human herpesviruses were significantly higher in the subjects with AD pathology than the subjects in the controls. Notably, a retrospective cohort study [10], following 33,448 people in Taiwan, suggested that the people who experienced herpesvirus infections had a 2.5-fold increased risk in developing AD compared to the people without the infections. However, these data are correlative and do not demonstrate a direct causal relationship between HSV-1 and AD. Thus, there is a further demand for revealing the causal relationship between the HSV-1 infection and the onset and progression of AD pathology.

Current understanding of the mechanisms regulating HSV-1 infections and Aβ Associated neuropathogenesis comes primarily from small-animal models, such as mice. However, wild-type mice don’t intrinsically develop AD-associated neuropathological phenotypes. Thus, the study of the Aβ associated neuropathology mainly relies on the transgenic mouse models that overexpress pivotal risk genes of family AD (FAD) [11]. For example, the 5 × FAD transgenic mouse is a widely-recognized AD mouse model that possesses a total of five typical familial mutations in amyloid precursor protein (*APP*) and presenilin 1 (*PSEN1*) genes discovered in the FAD patients. Using this model, Giovanna De Chiara and colleagues [12] found that HSV-1 infection accelerated Aβ deposition in the brain. The follow-up studies [13,14] further revealed that Aβ oligomers aggregated around the herpesvirus particles in the cortex, implying that Aβ oligomers may act as a type of endogenous antiviral protein in the innate immune system. However, the transgenic mouse models could not faithfully mimic the pathogenesis of AD that do not involved a genetic background of familial mutations. Besides, there are distinct neurophysiological differences between the human and the mouse brains across the multiple bio-hierarchical levels at the varied neurodevelopmental stages, including morphogenesis of cortex, cytoarchitecture of cortex, neuron polarization of neuroepithelium, and self-organization of cerebral cortex. Therefore, the transgenic mice are not faithful research models to study the human-specific neuropathophysiological characteristics in the late-onset AD patients.

Development of human physiologically relevant infection models is vital to understanding how virus infections impact the onset and progression of AD. Fortunately, emerging cerebral organoids offer a more human relevant in-vitro model to study the neurophysiology and disease of human cerebral cortex [15]. Specifically, cerebral organoids could emulate the human cerebral cortex’s cellular diversity [16], such as the intermediate progenitors and the neurogenic outer radial glia. Furthermore, the cerebral organoids can resemble the specific cytoarchitecture in the human cerebral cortex, including the cortical lumen organization [17] and cortical layers [18]. Recently, the cerebral organoids have been used to model neurodegenerative diseases [19] and neurotropic virus infections [20]. Cesar Gonzalez and his colleagues established cerebral organoid models derived from FAD patients’ induced pluripotent stem cells, and these models recapitulated some typical neuropathological features in the AD-patient brains, including Aβ accumulation, p-tau aggregation, and cellular apoptosis [21]. Moreover, in our previous study, we generated the HSV-1-infected cerebral organoids to reconstitute the neuropathological features of the neurodevelopmental disorders in the human fetal brain [22]. Besides, Leonardo D’Aiuto et al. used cerebral organoids to study the acute and latent HSV-1 infection, providing substantial evidence that the cerebral organoids are suitable to model HSV-1-CNS interactions [23].

In this study, we generated human-physiologically related cerebral organoids derived from human embryonic stem cells (hESCs) to investigate the influence of HSV-1 infection on the Aβ associated neuropathology. We found that the HSV-1-infected cerebral organoids could model typical neuropathological features associated with AD including multicellular Aβ deposition and dysregulated exogenous AD mediators. Notably, the HSV-1 infected cerebral organoids also demonstrated reactive gliosis, neuroinflammation, and neuron loss. Furthermore, we discovered that both Ribavirin (RBV) and Valaciclovir (VCV) significantly suppressed viral replication and rescued the HSV-1-induced Aβ associated neuropathology, implying their therapeutic effects on AD patients. Taken together, we developed effective human brain models for mimicking the HSV-1-induced Aβ associated neuropathology and further supported the AD viral hypothesis. We expected this model will facilitate the applications to explore therapeutic intervention and target HSV viral reservoirs relevant to AD.

## 2. Results

### 2.1. HSV-1 Led to Aβ Deposition in the Cerebral Organoids

**Figure 1:**
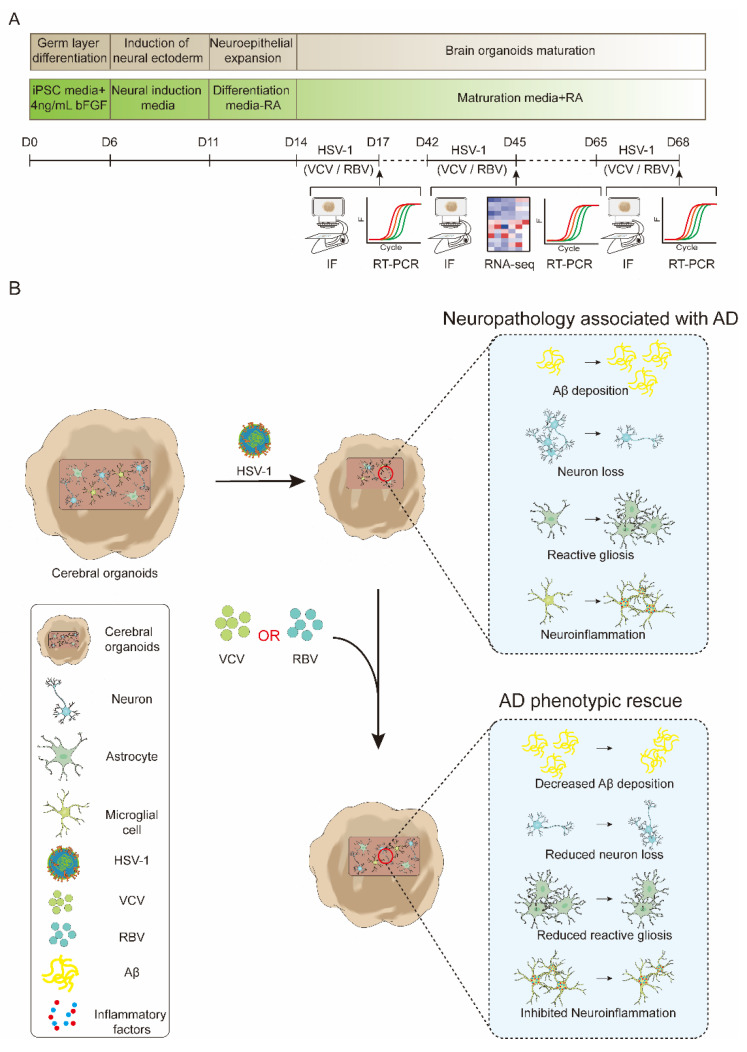
Schematic diagram of cerebral organoids for modeling of HSV-1-induced-Aβ associated neuropathology and phenotypic rescue. (**A**) Schematic procedure of HSV-1-infected cerebral organoids timing and method. (**B**) Cerebral organoids for modeling of HSV-1-induced-multiscale neuropathology associated with Alzheimer’s disease and phenotypic rescue. HSV-1-infected cerebral organoids could model typical neuropathological features associated with AD including multicellular Aβ deposition, neuron loss, reactive gliosis, and neuroinflammation. Furthermore, both RBV and VCV significantly rescued the HSV-1-induced pathological phenotypes associated with AD.

**Figure 2:**
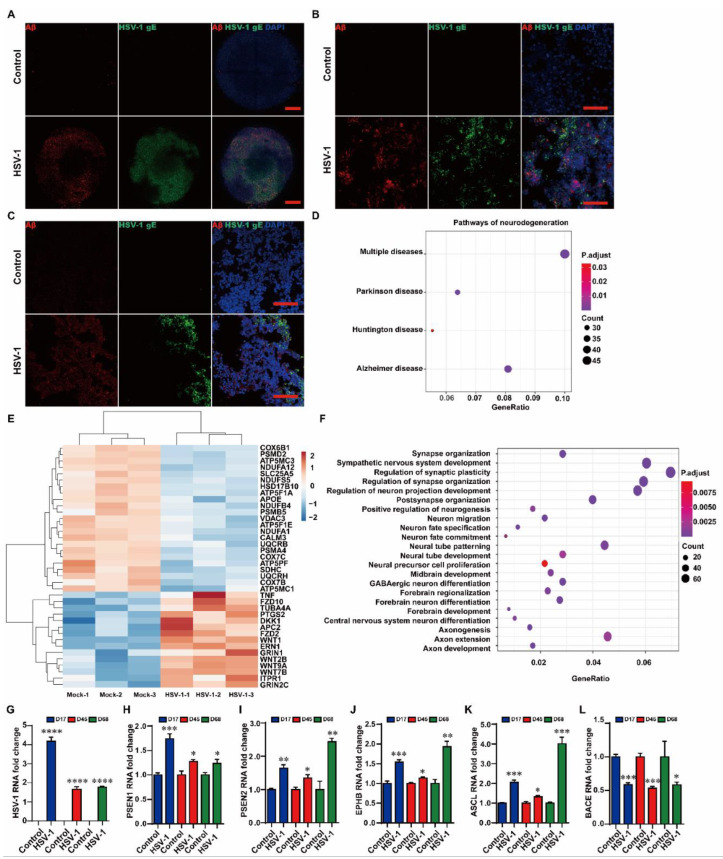
HSV-1 led to Aβ deposition in the cerebral organoids. (**A**) Whole-mount 3D immunofluorescence imaging of HSV-1 and Aβ in the cerebral organoids with or without HSV-1 infection at D14 + 3. Scale bars (red rectangle): 250 µm. (**B**,**C**) The immunostaining for the HSV-1 gE-positive and Aβ-positive cells in the cerebral organoids with or without HSV-1 infection at D42 + 3 and D65 + 3. Scale bars (red rectangle): 100 µm. (**D**,**E**) KEGG pathways enriched in the neurodegenerative diseases and the AD-related dysregulated genes in the cerebral organoids infected with the HSV-1, compared to the control. (**F**) Dot plot of selected enriched GO terms for the biological process for dysregulated genes in the cerebral organoids infected with the HSV-1, compared to the control. (**G**–**L**) RT-PCR analysis to monitor the mRNA expressions of *HSV-1, PSEN1, PSEN2, EPHB, ASCL,* and *BACE* in the HSV-1 infected cerebral organoids at D14 + 3, D42 + 3, and D65 + 3. The expression value was normalized to the *GAPDH* expression level. Data represent the mean ± SEM. * *p* < 0.05 vs. the control group, ** *p* < 0.01 vs. the control group, *** *p* < 0.005 vs. the control group, **** *p* < 0.001 vs. the control group, (*n* = 6).

To generate human cerebral organoids, we followed the protocol described by Lancaster and Knoblich. Cerebral organoids develop through intrinsic self-organizing processes. We found that cerebral organoids contained a well-defined ventricular zone (VZ) and cortical plate (CP) layers at D45. Specifically, the majority of the sites showed PAX6 (dorsal forebrain marker) positive cells on the interior of the neural-tube-like structures, surrounded by TUJ positive (newborn neuron marker) cells on the outer edge of the structures (Figure S1A). At day 68, late-born SATB2 positive neurons formed a layer partially separated from the early-born CTIP2 positive layer, suggesting the specification of upper and deep cortical layers (Figure S1B). To investigate the susceptibility of human brain cells to HSV-1 infection, we tested cerebral organoids generated via the previous protocols. The cerebral organoids at the different growth stages (day14, D14; day 42, D42; day 65, D65) were inoculated with HSV-1 for three days (D14 + 3, D42 + 3, D65 + 3), then we examined the cerebral organoids after inoculum removal (Figure 1A,B). The quantitative 3D immunofluorescence imaging of cerebral organoids revealed that HSV-1 infection increased the Aβ expression at D14 + 3 (Figure 2A), the results also showed that HSV-1 infection induced the Aβ deposition at D42 + 3 (Figure 2B) and D65 + 3 (Figure 2C).

The RNA sequencing (RNA-seq) analysis was also conducted to examine the regulatory effects of HSV-1 on the AD-associated gene networks in the cerebral organoids at D42 + 3. The KEGG pathway enrichment analysis (FDR < 0.05) indicated that the regulation of gene networks in the HSV-1-infected cerebral organoids were closely linked to neurodegenerative diseases, especially for AD (Figure 2D). The heat map (Figure 2E) further indicated that 30 AD-associated genes (e.g., *WNT, TNF,* and *APOE*) [24,25,26] were highly responsive to HSV-1 infection, predicting that HSV-1 infection may play an essential regulatory role in AD pathogenesis. The GO enrichment analysis of the differentially expressed genes (Figure 2F) showed that the highly enriched categories were related to synapse organization, synapse assembly, neural tube development, nervous system development, axon guidance, cell morphogenesis neuron differentiation, neuron projection guidance, and neuron projection development. The remarkable convergence of KEGG pathway enrichment analysis and GO enrichment analysis prompted us to hypothesize that HSV-1 infection led to the AD-associated neuropathology in the cerebral organoids.

Then we determined the expression of widely recognized AD mediator genes, including *PSEN1, PSEN2, EPHB, ASCL,* and *BACE* in the cerebral organoids. The real-time PCR (RT-PCR) analysis indicated that HSV-1 infection caused up-regulation of *HSV-1, PSEN1, PSEN2, EPHB,* and *ASCL* at mRNA levels (Figure 2G–K) and down-regulation of *BACE* mRNA expression in the cerebral organoids (Figure 2L) at D14 + 3, D42 + 3, and D65 + 3, compared to the uninfected groups.

### 2.2. HSV-1 Infection Resulted in the Neuron Loss

**Figure 3:**
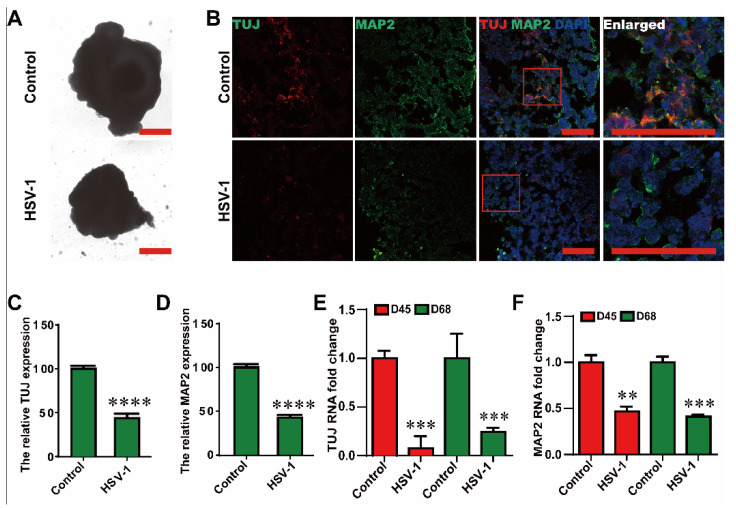
HSV-1 infection resulted in the neuron loss. (**A**) The bright field images of cerebral organoids with or without HSV infection (D65 + 3), the red frame line is the position of the enlarged figure. Scale bars (red rectangle): 1 mm. (**B**) Co-immunostaining of TUJ and MAP2 were identified by immunofluorescence in the HSV-1 infected cerebral organoids at D65 + 3, Scale bars (red rectangle): 100 µm. (**C**,**D**) The relative fluorescence intensity statistics of TUJ and MAP2 expressions were shown in different groups. (**E**,**F**) RT-PCR analysis to monitor the mRNA expressions of *TUJ* and *MAP2* in HSV-1-infected cerebral organoids at D42 + 3 and D65 + 3. The expression value was normalized to the *GAPDH* expression level. Data represent the mean ± SEM. ** *p* < 0.01 vs. the control group, *** *p* < 0.005 vs. the control group, **** *p* < 0.001 vs. the control group, (*n* = 6).

In order to test whether HSV-1 infection inhibits neuronal differentiation. We found that HSV-1 infection could reduce the size of cerebral organoids (D65 + 3) after three days of infection, suggesting neural loss (Figure 3A). Then, we examined neuronal differentiation hallmarks of different stages in the infection models. The HSV-1 infected cerebral organoids exhibited a loss in the neuronal-associated protein (TUJ and MAP2) by immunofluorescence (Figure 3B–D) at D65 + 3. In addition, the mRNA expressions of *TUJ* and *MAP2* in HSV-1-infected cerebral organoids were also examined by RT-PCR at D42 + 3 and D65 + 3 (Figure 3E,F), which were consistent with the results observed in immunofluorescence analysis, indicating a distinct neuron loss by HSV-1 infection.

### 2.3. HSV-1 Infection Led to Reactive Gliosis and Neuroinflammation in the Cerebral Organoids

**Figure 4:**
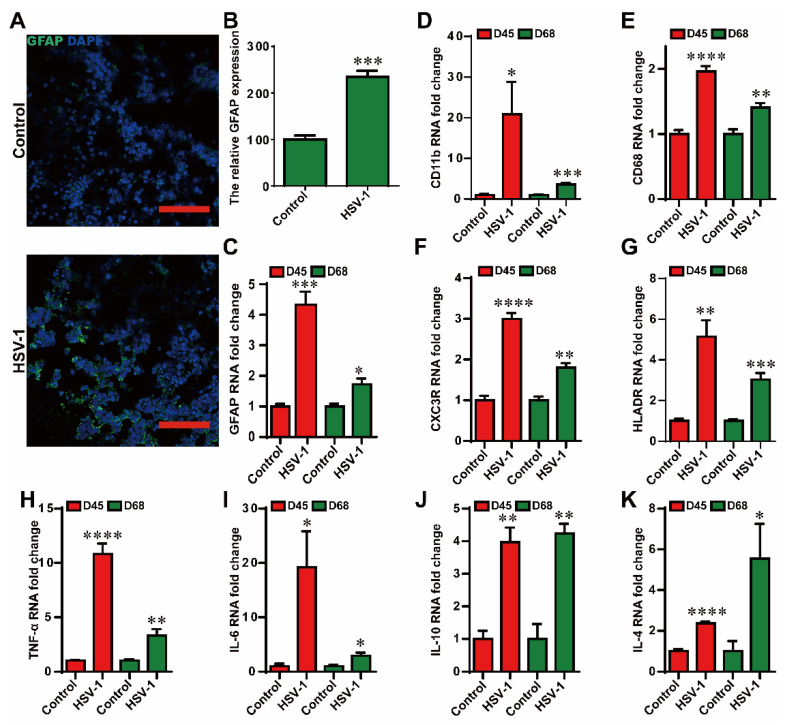
HSV-1 infection led to reactive gliosis and neuroinflammation in the cerebral organoids. (**A**,**B**) The expression of GFAP was identified by immunofluorescence staining, and the quantifications for relative fluorescence intensity statistics of GFAP expressions were shown. Scale bars (red rectangle): 100 µm. (**C**) The mRNA of *GFAP* was monitored by RT-PCR in the HSV-1 infected cerebral organoids at D42 + 3 and D65 + 3. (**D**–**G**) Validation by RT-PCR of microglia cells markers (*CD11b*, *CD68*, *CX3CR*, and *HLADR*) in cerebral organoids at D42 + 3 and D65 + 3. (**H**–**K**) The mRNA expressions were examined for the inflammatory cytokines (*TNF-α, IL-6, IL-10,* and *IL-4*) using RT-PCR in cerebral organoids in the different groups at D42 + 3 and D65 + 3. The expression value was normalized to the *GAPDH* expression level. Data represent the mean ± SEM. * *p* < 0.05 vs. the control group, ** *p* < 0.01 vs. the control group, *** *p* < 0.005 vs. the control group, **** *p* < 0.001 vs. the control group, (*n* = 6).

It is widely accepted that glial-mediated reactive gliosis and neuroinflammation contribute to the progression of AD. Thus we examined the reactive gliosis and neuroinflammation in the HSV-1 infected cerebral organoids. We assessed the reactive gliosis in the organoids using immunofluorescence staining at D65 + 3. The results indicated that the relative fluorescence intensity of GFAP (a marker of astrocytes) were significantly higher in the HSV-1-infected cerebral organoids compared to the control group (Figure 4A,B). In addition, the mRNA expressions of *GFAP* in HSV-1-infected cerebral organoids were also examined by RT-PCR at D42 + 3 and D65 + 3 (Figure 4C), which were consistent with the results observed in immunofluorescence analysis. CD11b and CD68 is a marker of microglia. The overexpression of CD11b and CD68 in active microglia is considered as the reactive gliosis in AD. What is more, the apparent increase in mRNA expressions of the activated microglia markers (*CD11b, CD68, CXC3R*, and *HLADR*) were observed in the cerebral organoids at D42 + 3 and D65 + 3 (Figure 4D–G). Next, we examined the neuroinflammatory responses in the cerebral organoids at D42 + 3 and D65 + 3. The HSV-1 infection induced increased mRNA expressions of the pro-inflammatory mediators (*TNF-α* and *IL-6*) and anti-inflammatory mediators (*IL-10* and *IL-4*) (Figure 4H–K). These observations demonstrated that HSV-1 infection led to reactive gliosis and neuroinflammation in the cerebral organoids.

### 2.4. RBV and VCV Treatments Resulted in Decreased Aβ Deposition and Normalized AD-Mediator Expression in the Cerebral Organoids

RBV, the synthetic guanosine analog, has been widely used as a broad-spectrum antiviral drug in clinical. In contrast, VCV is one of the most commonly used antiviral agents specifically targeting the HSV DNA replication.

**Figure 5:**
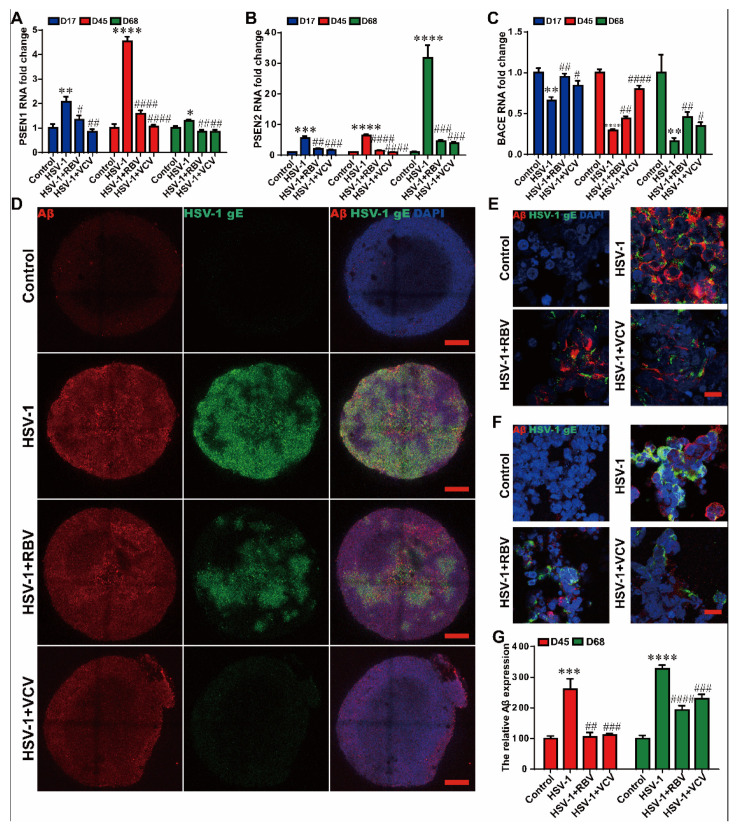
RBV and VCV treatments resulted in decreased Aβ deposition and normalized AD-mediator expression in the cerebral organoids. (**A**–**C**) The mRNA expressions of AD mediator genes (*PSEN1*, *PSEN2*, and *BACE*) were examined by RT-PCR in cerebral organoids in different groups at D14 + 3, D42 + 3 and D65 + 3. (**D**) Whole-mount 3D immunofluorescence imaging of HSV-1 and Aβ in the HSV-1-infected cerebral organoids treated with RBV or VCV at D14 + 3. Scale bars (red rectangle): 250 µm. (**E**,**F**) Immunocytochemistry analysis and (**G**) quantification of the HSV-positive and Aβ-positive cells in the HSV-1 infected cerebral organoids with the RBV or VCV treatment at D42 + 3 and D65 + 3. Scale bars (red rectangle): 100 µm. Data represent the mean ± SEM. * *p* < 0.05 vs. the control group, ** *p* < 0.01 vs. the control group, *** *p* < 0.005 vs. the control group, **** *p* < 0.001 vs. the control group, # *p* < 0.05 vs. the HSV-1 infection group, ## *p* < 0.01 vs. the HSV-1 infection group, ### *p* < 0.005 vs. the HSV-1 infection group, #### *p* < 0.001 vs. the HSV-1 infection group, (*n* = 6).

To investigate the effects of these two antiviral drugs on Aβ deposition and AD mediator gene expressions in cerebral organoids at the different growth stage D42 and D65, VCV or RBV was added concurrently with HSV-1 infection for 3 days. The AD mediator genes, including *PSEN1*, *PSEN2*, and BACE, have abnormal expressions in the HSV-1 infected-cerebral organoids at D42 + 3 and D65 + 3; both RBV and VCV effectively revised the HSV-1-induced changes of AD mediators (Figure 5A–C).

To further explore the therapeutic effects of the antiviral drugs on the Aβ deposition. The 3D immunofluorescence analysis showed that the Aβ deposition induced by HSV-1 infection was reduced in the both RBV and VCV treatments groups in the cerebral organoids at D42 + 3 (Figure 5D). Concomitantly, we observed a similar reduction in HSV-1 Aβ deposition under the treatment of RBV or VCV in the cerebral organoids at D42 + 3 and D65 + 3 compared to the non-treatment group (Figure 5E,F). Quantitative image analysis (Figure 5G) displayed that the Aβ expression was increased in the HSV-1 infection group, while the Aβ expression was significantly suppressed by both the RBV and the VCV treatments. Together, these observations provide strong preclinical evidence for the therapeutic effects of the RBV and the VCV on the HSV-1-induced Aβ deposition and aberrant AD mediator expressions.

### 2.5. RBV and VCV Treatments Reduced Reactive Gliosis and Neuroinflammation in the Cerebral Organoids

**Figure 6:**
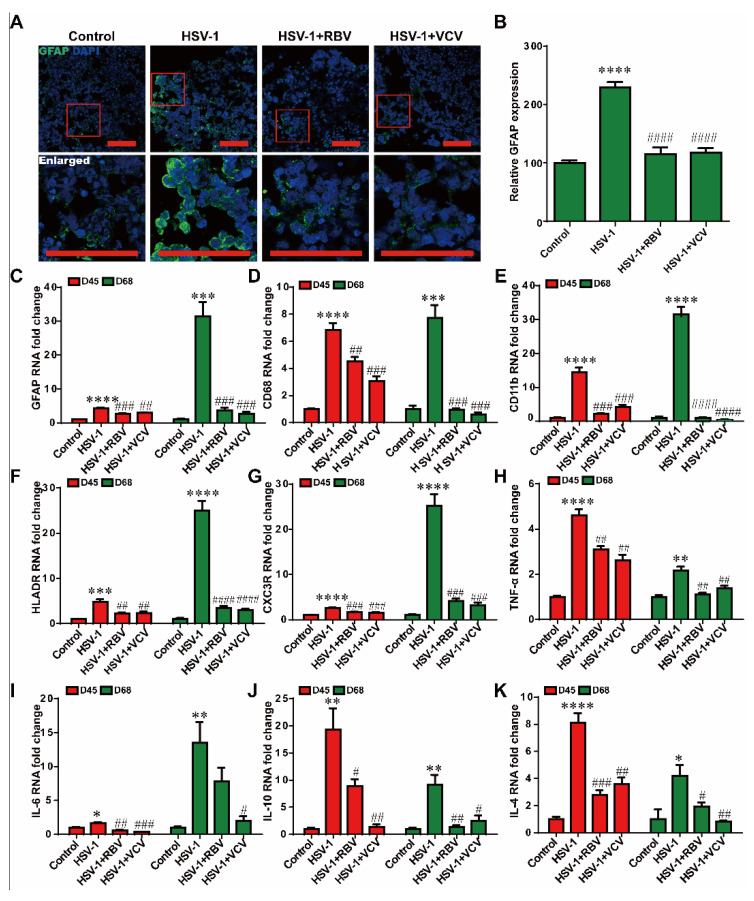
RBV and VCV treatments reduced reactive gliosis and neuroinflammation in the cerebral organoids. (**A**) The expression of GFAP was identified by immunofluorescence staining, and the quantifications (**B**) for relative fluorescence intensity statistics were shown, the red frame line is the position of the enlarged figure. Scale bars (red rectangle): 100 µm. (**C**) The mRNA expression of astrocyte cell marker (*GFAP*) was examined by RT-PCR in cerebral organoids in the different groups at D42 + 3 and D65 + 3. (**D**–**G**) The mRNA expression of microglia cell markers (*CD11b*, *CD68*, *HLADR*, and *CXC3R*) and astrocyte cell marker (*GFAP*) were examined by RT-PCR in cerebral organoids in the different groups at D42 + 3 and D65 + 3. (**H**–**K**) The mRNA expressions were quantified for the pro-inflammatory (*TNF-α* and *IL-6*) and anti-inflammatory (*IL-10* and *IL-4*) mediators. Data represent the mean ± SEM. * *p* < 0.05 vs. the control group, ** *p* < 0.01 vs. the control group, *** *p* < 0.005 vs. the control group, **** *p* < 0.001 vs. the control group, # *p* < 0.05 vs. the HSV-1 infection group, ## *p* < 0.01 vs. the HSV-1 infection group, ### *p* < 0.005 vs. the HSV-1 infection group, #### *p* < 0.001 vs. the HSV-1 infection group, (*n* = 6).

To validate the inhibition of reactive gliosis and anti-neuroinflammation effectivity of these two antiviral drugs in the HSV-1 infected cerebral organoids. We incubated VCV or RBV concurrently with HSV-1 infected cerebral organoids of different stages (D42 and D65) for 3 days. Our results indicated that the expression of astrocyte marker GFAP was significantly higher in the HSV-1-infected cerebral organoids compared to the organoids in the control group. Furthermore, the HSV-1-induced overexpression of GFAP were significantly suppressed in the RBV and the VCV treatment groups by the immunofluorescence staining and quantitative image analysis (Figure 6C), compared to those expression in the HSV-1 infection group, suggesting the therapeutic roles of RBV and VCV in the HSV-1-induced gliosis. Concomitantly, we observed that RBV and VCV treatments decreased the expressions of microglia markers (*CD11b, CD68, HLADR,* and *CXC3R*) at mRNA levels in contrast to the results obtained with HSV-1-infected groups (Figure 6D–G). The RBV and the VCV treatment successfully rescued the HSV-1-induced gliosis. Next, we examined the neuroinflammatory responses in the HSV-1 infected cerebral organoids. As shown in Figure 6H–K, HSV-1 infection strongly increased mRNA expressions of pro-inflammatory mediators (*TNF-α* and *IL-6*) and anti-inflammatory mediators (*IL-10* and *IL-4*), which were evidently turned down by RBV or VCV treatment.

### 2.6. RBV and VCV Treatments Reduced the Neuron Loss

**Figure 7:**
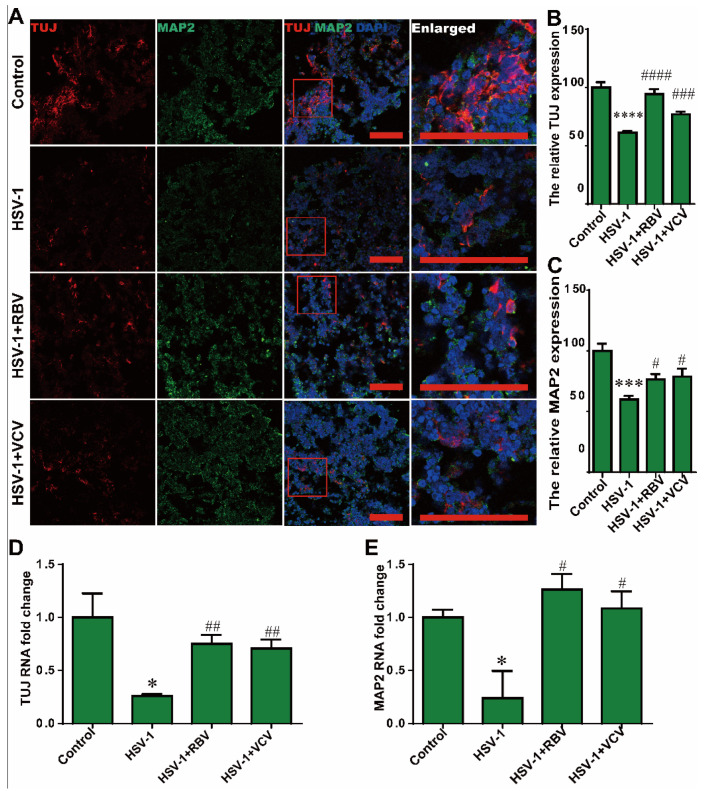
RBV and VCV treatments reduced the neuron loss. (**A**) Co-immunostaining with TUJ and MAP2 in cerebral organoids in the different group at D65 + 3, the red frame line is the position of the enlarged figure. Scale bars (red rectangle): 100 µm. (**B**,**C**) The relative fluorescence intensity statistics of TUJ and MAP2 expressions in cerebral organoids were shown in different groups at D65 + 3. (**D**,**E**) The *TUJ* and *MAP2* mRNA expressions were quantified by RT-PCR in the cerebral organoids in the different groups at D42 + 3 and D65 + 3. Data represent the mean ± SEM. * *p* < 0.05 vs. the control group, *** *p* < 0.005 vs. the control group, **** *p* < 0.001 vs. the control group, # *p* < 0.05 vs. the HSV-1 infection group, ## *p* < 0.01 vs. the HSV-1 infection group, ### *p* < 0.005 vs. the HSV-1 infection group, #### *p* < 0.001 vs. the HSV-1 infection group, (*n* = 6).

To further demonstrate the neuroprotective effects of these two antiviral drugs in the HSV-1 infected cerebral organoids. We also investigated the therapeutic effects of RBV and VCV on neuron loss. Strikingly, immunofluorescence analysis revealed that the cerebral organoids displayed a markedly decrease in the expressions of TUJ and MAP2 after HSV-1 infection at D65 + 3 (Figure 7A–C), and both the RBV and the VCV treatments had the recovery effect on the neuron fate commitment. Similarly, the HSV-1-infected cerebral organoids exhibited a low level of *TUJ* and *MAP2* (Figure 7D,E) mRNA expression, the RBV and the VCV treatments reversed HSV-1-induced the inhibition of neuronal differentiation. These results reveal that RBV or VCV treatments reversed HSV-1-induced neuron loss.

## 3. Discussion

Recently, there are increasing clinical evidence [27] and basic researches [28,29] indicating that there is strong correlation linking HSV-1 infection with Aβ Associated pathogenesis, which supports the AD viral hypothesis [30]. With the limited access to human brain tissue, the further mechanistic verification of this correlation is highly relying on the AD transgenic mouse models [12]. However, HSV-1 is a highly human-specific virus and does not infect the wild type mice in the natural settings [31]. In addition, these transgenic mouse infection models fail to fully capture the human-specific neuropathological features due to the distinct differences between humans and mice in terms of polarized neuroepithelium, intricate neurodevelopmental trajectory, and spatiotemporal self-organization in the cerebral cortex. Thus, there is a strong demand to develop more persuasive preclinical models for studying the casual relation between Aβ associated neuropathology and HSV infection. Recent advance in human cerebral organoids offers a more faithful human-relevant model system to examine the relationship between Aβ associated neuropathology and HSV-1. In this work, we used cerebral organoids to investigate the influence of HSV-1 infection on Aβ associated neuropathology. We found that HSV-1-infected cerebral organoids could model Aβ associated neuropathology across the genetic, cellular, and tissue levels, including the Aβ deposition, dysregulated AD mediators, reactive gliosis, neuroinflammation, and impaired neuronal differentiation, which were associated AD. Furthermore, we discovered that both RBV and VCV significantly reduced HSV-1 replication and rescued these HSV-1-induced multiscale pathologic phenotypes. Our work intended to provide high-fidelity human brain infection models to evaluate the essential roles of HSV-1 infection on Aβ associated neuropathology, with the application in identifying the effects of drug candidates.

The Aβ deposition is a typical hallmark pathology of AD. In our study, we demonstrated that HSV-1 infection resulted in the Aβ deposition in the cerebral organoids, which was reminiscent of amyloid plaques found in AD patients. In general, the Aβ homeostasis of in human brain is determined by the three kinetic factors: (i) the Aβ generation, (ii) the transport of Aβ across the BBB, and (iii) the Aβ degradation in the brain parenchyma [32]. The AD mediator genes closely regulate these three kinetic factors in the brain. For example, the β-secretase (BACE) and γ-secretase subunits (PSEN1 and PSEN2) are responsible for the Aβ generation [33]. APOE, the only cholesterol transporter in the brain, participates in both the Aβ transport across the BBB [34] and the Aβ degradation [32]. Notably, a recent Bordeaux-3C prospective cohort study [35] assessed the 10-year risk of AD in 1037 participants and found that the APOE4 carriers with frequent reactivations of HSV-1 had a three-fold increased risk of AD, suggesting the interaction between *APOE* gene and HSV-1 in AD. However, it is not clear whether HSV-1 may influence the AD mediator genes in human brain. In our study, we found the AD mediator genes *PSEN1*, *PSEN2*, *ASCL*, and *EPHB* in the cerebral organoids were significantly upregulated, meanwhile the BACE expression was decreased in response to the HSV-1 infection. These findings for the first time clarified that the HSV-1 infection influence the AD mediator genes in the cerebral organoids, implying the causative role of HSV-1 in AD pathogenesis.

The inability to faithfully recapitulate pathophysiological features of human diseases in the traditional cell culture and animal models causes that most preclinically-validated drug candidates failed in the clinical trials. Despite enormous academic and commercial research investments, an effective treatment for AD remains elusive. Specifically, in the pipeline of AD drug discovery, 99.6% of AD candidate drugs that showed the promising results in the traditional models failed in clinical trials [36,37]. Currently, only five treatment options have been approved to treat the cognitive symptoms of AD, but these treatments have only modest benefit on cognition and no effect on reversing the disease progression [38,39]. Here, we discovered that candidate drugs RBV and VCV had the ability to rescue Aβ associated neuropathology in the HSV-1 infected cerebral organoids. VCV, targeting to herpes simplex and herpes zoster, is approved as the antiviral medication for the infections of HSV-1, HSV-2, herpes zoster, and chickenpox. Recently, VCV is repurposed as the first-ever anti-AD drug in phase II clinical trial where 130 mild AD patients (MMSE range 20–28) with positive HSV-1 or HSV-2 serum antibodies are undergoing an 18-month, randomized, double-blind, placebo-controlled trial [40]. Furthermore, a recent retrospective cohort study [10] performed in Taiwan suggested that VCV could attenuate the risk of dementia in patients with HSV infections. In this study, we found that this anti-HSV drug could significantly repair the HSV-1-induced Aβ associated neuropathology in the cerebral organoids, including the dysregulated AD mediators, impaired neuronal differentiation, abnormal microglial activation, and neuroinflammation. Together with the retrospective cohort study, we consider VCV is a promising AD candidate drug and recommend to accelerate its clinical trials.

Ribavirin (RBV) is a broad-spectrum antiviral drug [41] against a wide range of DNA and RNA viruses. RBV has been introduced to treat chronic hepatitis C virus (HCV) infection [42] for decades. What is more, RBV is repurposed candidate drug for cancer treatment [43]. In our result, RBV rescued the AD-related neuropathological phenotypes associated with AD in the cerebral organoids. Our findings strengthen the potency of RBV as a candidate or part of drug combination therapy for the AD treatment.

Recently, emerging human physiologically relevant in-vitro organotypic and histotypic models offer exciting alternative platforms for the basic and preclinical researches in the biomedical field. These models bridge the gaps between the single cell level and the organismal level in the traditional biological model systems and potentially provide more accurate understanding for human diseases and faithful evaluation for drug candidates. To establish these models, organoids and microfabricated scaffolds represent two critical but distinct approaches based on the strategies of developmental biology and bioengineering, respectively. Organoids allow generating miniaturized organs from stem cells in vitro by mimicking organogenesis at the human embryonic development stage. In contrast, the microfabricated scaffolds generate faithful tissue-like structures by reverse engineering and bionic fabrication using bioactive materials and living cells. These two models have demonstrated great potential to address the long-lasting challenge in elucidating the ultimate etiology and the underlying mechanism of AD onset and progression. For example, Dana M. Cairns et al. [44] pioneered a 3D bioengineered brain model by seeding NSCs into the silk protein scaffold based microdevice to study the effects of HSV-1 infection on AD. Using this bioengineered brain model, they found HSV-1 infection led to the formation of AD-like phenotypes, including the Aβ formation, gliosis, neuroinflammation, and diminished neural network functionality. However, this model could not mimic the cortical cellular diversity and spatiotemporal self-organization in the human cerebral cortex. The apparent cortical structure changes and cortical dysfunctions are indeed critically neuropathological features in AD patients [45,46]. The cerebral organoids and the bioengineered brain models can perfectly supplement each other with their benefits. The cerebral organoids explore the power of developmental biology to reproduce the different stages of brain development, especially, the spatiotemporal self-organization cerebral cortex.

In summary, our study examined the effects of HSV-1 infection on the cerebral organoids and found that the HSV-1 infection causes multiscale neuropathology associated with AD across the multiple bio-hierarchical levels at the different neurodevelopmental stages. Furthermore, we discovered that antiviral drugs RBV and VCV have potential value in the onset and progression of AD. Overall, our study supports the rationality of the AD viral hypothesis and identifies RBV and VCV as promising drug candidates for AD treatments. Further efforts towards building better HSV-1-infected brain models will be to build brain organoid-on-a-chip, which may include blood-brain barrier, multiscale vascular structure, and precise neuro-microenvironment control over organoids on a microfabricated scaffold device by exploring the benefits from both developmental biology and bioengineering. We expect these new models will offer superior high-fidelity to study the viral mechanisms in neurodegenerative diseases, screen potential drug therapies, and aid precision medicine in the future.

## 4. Materials and Methods

### 4.1. Generation of Human Cerebral Organoids

The human cerebral organoids were generated from H9 hESCs using a previously reported protocol with minor modification [20]. Detailed composition of cerebral organoid differentiation medium was described in the Table 1. Briefly, Accutase (A1110501, Gibco, CA, USA) was used to disassociate the hESCs into single cells. A total number of 2000 cells were then plated into the hanging drop culture plates (InSphero AG, Schlieren, Switzerland) to form the single embryoid body (EB) in the first 24 h, and then the EBs were transferred to the ultra-low-attachment 96-well plates (7007, Corning, New York, NY, USA) in the EB formation medium with 4 ng/mL bFGF (100-18B, Peprotech, NJ, USA) and 50 mM ROCK inhibitor Y27632. The EBs were fed every other day for 6 days then transferred to the low adhesion 24-well plates in the induction media. On day 12, the EBs were embedded in the seated Matrigel droplets (356231, Corning, New York, NY, USA), and these droplets were solidified at 37 °C. Embedded EBs were subsequently cultured in the neural expansion medium. The embedded EBs were cultured in the stationary condition in 6-well plates for 4 days. Then the plates were transferred to an orbital shaker (HS-25A, MIULAB, Hangzhou, China) rotating continuously at 75 rpm. The culture medium in the dishes was replaced with the neural maturation medium.

### 4.2. HSV-1 Infection and Phenotypic Rescue

HSV-1 strain F stocks were propagated in the Vero cells (African green monkey kidney), with the DMEM supplemented with 2% (*v*/*v*) Fetal Bovine Serum (FBS). The propagation was terminated when 90% of the cells appeared with the cytopathic effect. The virus stocks were harvested by repeatedly freezing and thawing the supernatants and the cells for three times, followed by filtering with the 0.22 µm filter and stored in the −80 °C fridge before use. The plaque assay determined the titers of HSV-1 stocks in the Vero cells. Briefly, the Vero cells were cultivated in 6-well plates and incubated with a series of 10-fold virus dilutions for two hours. Then cells were overlaid with 2 mL/well of DMEM (11054020, Gibco) containing 1.2% (*v*/*v*) methylcellulose, 10% (*v*/*v*) FBS, 1% (*v*/*v*) penicillin-streptomycin solution (P0781, Sigma). Plates were incubated at 37 °C with 5% CO_2_ for five days. Cells were fixed with 4% (*w*/*v*) paraformaldehyde (PFA) and stained with 0.5% (*w*/*v*) crystal violet solution. HSV-1 strain F stocks were maintained at a stock concentration of 10^7^ pfu/mL. The cerebral organoids at the different developmental stages (D14 + 3; D42 + 3; D65 + 3) were incubated with the titer of HSV-1(100,000 pfu/organoid) in the culture medium for three days. For the mock infections, an equal volume of control culture medium was used. To investigate the effects of VCV and RBV on the pathological phenotypes of cerebral organoids, VCV or RBV was added at a concentration of 200 μM concurrently with HSV-1 infection. After an additional three days’ incubation, the organoids were completely washed with the neural maturation medium.

### 4.3. Histology and Immunofluorescence

Immunofluorescence analysis was conducted as a previous report described [47]. To perform immunofluorescence analysis, cerebral organoids were fixed in 4% (*w*/*v*) PFA and sectioned into 10 µm thick slices with a cryostat (CM1950, Leica, Wetzlar, Germany). Images were captured via a Leica TCS SP8 STED confocal microscope equipped with a LAS X software. Detailed antibody staining was described in the Table 2. The same laser intensity, detector sensitivity, amplification value, and offset were used for all micrograph acquisitions for quantification of the same protein expression. The expression level of nuclear and cytosolic proteins was calculated by the following formula.

(1) $$\mathrm{The}\;\mathrm{protein}\;\mathrm{expression}\;\mathrm{level}=\frac{\mathrm{Integrated}\;\mathrm{optical}\;\mathrm{density}\;\left(\mathrm{IOD}\right)}{\mathrm{The}\;\mathrm{total}\;\mathrm{area}}$$

Moreover, each IOD/area value was calculated by subtracting background IOD/area value from the directly measured IOD/area value. For each organoid, integrated optical density (IOD)/area was measured in each of five randomly consecutive fields (40×) in each of three slides through LAS X software (LAS X, Wetzlar, Germany). For each field, areas of interest (periodontal tissues) were selected for the measurement of IOD and area. Moreover, each IOD/area value was calculated by subtracting background IOD/area value from the directly measured IOD/area value. Then, the mean value of these 15 fields was used as the expression level of target protein for that organoid [48]. Images analysis was conducted with NIH Image J (NIH, Bethesda, MD, USA) and LAS X software.

### 4.4. Whole-Mount Immunostaining of Organoids

We performed whole-mount immunostaining followed by confocal microscopy to examine the localizations and organization of the HSV-1-induced Aβ deposition within the cerebral organoids. The organoids were washed with 1 × PBS and fixed overnight in 4% (*v*/*v*) PFA at 4 °C. After being washed with PBS for five hours, the organoids were blocked overnight at RT in 0.5% (*v*/*v*) BSA and 0.125% (*v*/*v*) Triton X-100 in PBS. The organoids were incubated in the primary antibodies (anti-HSV-1 gE and anti-Aβ) diluted in 0.5% (*v*/*v*) BSA and 0.125% (*v*/*v*) Triton-100 in 1 × PBS for two days at 4 °C. Unbound antibodies were removed via multiple washes with 1 × PBS for one day at RT. Then, the cerebral organoids were then incubated with Alexa Fluor 568 Goat anti-Mouse (1:200 dilution, Life Technologies, Massachusetts, USA), Alexa Fluor 488 Donkey anti-Rabbit (1:200 dilution, Life Technologies) and Donkey anti-Rabbit (1:200 dilution, Life Technologies) for four hours following nuclei staining with DAPI (1:1000 dilution) for two hours. 3D image stacks (100 µm in the thickness) were acquired for representative organoids. The interval between the neighboring stacks was 4 µm.

### 4.5. RT-PCR

Total mRNAs were isolated from the cerebral organoids or the cells using Trizol, then cDNA was synthesized using ABScript III RT Master Mix (RM21452, ABclonal Technology, Wuhan, China). RT-PCR was performed using SYBR Green Real-time PCR Master Mix (RK21203, ABclonal Technology) under the following reaction conditions (35 cycles): denaturation at 95 °C for 1 min, annealing at 58 °C for 30 s, and extension at 72 °C for 30 s. Primer sequences were described in Table 3. The expression levels were normalized relative to the expression of the housekeeping gene *GAPDH* using the comparative Ct–method 2^−ΔΔCt^.

### 4.6. RNA-Seq and Data Analysis

The PSC-derived cerebral organoids were infected with the HSV-1(1:100,000 pfu/organoid). The inocula were removed after three days of infection. The cerebral organoids were harvested, and the RNAs were extracted. RNA sequencing was performed using an Illumina NextSeq 6000 with an average of 20 million reads per run. The Gene Ontology (GO) enrichment analysis, Kyoto Encyclopedia of Genes and Genomes (KEGG) pathway enrichment analysis was performed using DAVID database (accessed on 1 December 2020). In order to get astringent DEG data set, only DEG with ≥1.5-fold change was used for GO enrichment analysis and KEGG pathway enrichment analysis. The transcriptomics data have been submitted to the Sequence Read Archive with the database identifiers PRJNA759081.

### 4.7. Statistical Analysis

Statistical analysis of data was expressed as means ± SEM. Student’s *t*-tests were applied to data with equal or more than two groups. ANOVA analysis was used for comparing data with greater than two groups. In all the analyses, group differences were considered statistically significant as follows: * *p* < 0.05, ** *p* < 0.01, *** *p* < 0.005, **** *p*< 0.001. Sample sizes were indicated in the figure legends.

## Figures and Tables

**Table 1:** Composition of Cerebral organoid differentiation medium.

Medium Name	Component	Supplier	Identifier	Volume or Concentration
EB formation medium	mTeSR1	Stem Cell Technologies, Vancouver, BC, Canada	05850	50 mL
	bFGF	Peprotech	100-18B	4 ng/mL
	ROCK inhibitor Y27632	Stem Cell Technologies	72304	50 μM
Induction medium	DMEM/F12	Gibco	11330032	50 mL
	N2 supplement (100×)	Invitrogen, MA, USA	17502048	1×
	Non-Essential Amino Acids (100×)	Gibco	11140050	1×
	GlutaMAX (100×)	Gibco	35050061	1×
	Heparin	Sigma	H3149	1 μg/mL
Neural expansion medium	DMEM/F12	Gibco	11330032	24 mL
	Neurobasal medium	Gibco	21103049	24 mL
	N2 supplement (100×)	Gibco	17502048	0.5×
	B27 supplement—Vitamin A (50×)	Gibco	12587010	0.5×
	GlutaMAX (100×)	Gibco	35050061	1×
	Non-Essential Amino Acids (100×)	Gibco	11140050	0.5×
	Human insulin	Sigma-Aldrich, MO, USA	I9278-5ML	2.5 µg/mL
	beta-mercaptoethanol	Merck, NJ, United States	8057400005	25 nM
Neural maturation medium	DMEM/F12	Gibco	11330032	24 mL
	neurobasal medium	Gibco	21103049	24 mL
	N2 supplement (100×)	Gibco	17502048	0.5×
	B27 supplement + vitamin A (50×)	Gibco	17504044	0.5×
	GlutaMAX (100×)	Gibco	35050061	1×
	Non-Essential Amino Acids (100×)	Gibco	11140050	0.5×
	Human insulin	Sigma-Aldrich	I9278-5ML	2.5 µg/mL
	beta-mercaptoethanol	Merck	8057400005	25 nM

**Table 2:** Antibodies used in immunofluorescence.

Antibodies	Dilution	Source	Identifier
HSV-1 gE	1:200	Abcam, Cambridge, UK	Ab6510
Amyloid-β_1–42_ (Aβ)	1:200	Abcam	ab10148
MAP2	1:200	Abcam	ab11267
TUJ	1:200	Abcam	ab7751
TUJ	1:200	ABclonal Technology	A17913
GFAP	1:200	Invitrogen	MA5-12023

**Table 3:** Primer sequences used for RT-PCR.

Genes	Forward Primers	Reverse Primers
*HSV-1*	CTGCACGCACATGCTTGCCT	CTCGGGTGTAACGTTAGACC
*PSEN1*	TGGCTACCATTAAGTCAGTCAGC	CCCACAGTCTCGGTATCTTCT
*PSEN2*	CTGACCGCTATGTCTGTAGTGG	CTTCGCTCCGTATTTGAGGGT
*BACE*	CCATCCTTCCGCAGCAATA	CGTAGAAGCCCTCCATGATAAC
*ASCL1*	AACAGTCAACCAACCCCATC	GCTGTGCGTGTTAGAGGTGA
*TUJ*	TGATGCGGTCGGGATACTC	TGGGCCAAGGGTCACTACAC
*MAP2*	CAGGAGACAGAGATGAGAATTCC	CAGGAGTGATGGCAGTAGAC
*CD11b*	CCAGAGAATCCAGTGTGA	GTTATGCGAGGTCTTGATG
*CD68*	CTTCTCTCATTCCCCTATGGACA	GAAGGACACATTGTACTCCACC
*HLADR*	CCCAGGGAAGACCACCTTT	CACCCTGCAGTCGTAAACGT
*CX3CR1*	CTTACGATGGCACCCAGTGA	CAAGGCAGTCCAGGAGAGTT
*GFAP*	ACTGGCAGAGCTTGTTAGTG	AGTGACAGGAAGAGGTGAGA
*TNF-α*	GTGAGGAGGACGAACATC	GAGCCAGAAGAGGTTGAG
*IL-6*	TGAGAGTAGTGAGGAACAAG	CGCAGAATGAGATGAGTTG
*IL-10*	TGGAGCAGGTGAAGAATG	TCTATGTAGTTGATGAAGATGTC
*IL-4*	CCTCTGTTCTTCCTGCTA	AGATGTCTGTTACGGTCAA
*GAPDH*	GGACCTGACCTGCCGTCTAG	GTAGCCCAGGATGCCCTTGA

## Data Availability

All data that support the findings of this study are included within the article.

## Supplementary Materials

The following supporting information can be downloaded at: https://www.mdpi.com/article/10.3390/ijms23115981/s1.

## Abbreviations

AD, Alzheimer’s disease; HSV-1, Herpes simplex virus type I; FAD, Familial AD; Aβ, Amyloid β-protein; P-Tau, Phosphorylated tau; APP, Amyloid precursor protein; VCV, Valacyclovir; BBB, Blood-brain barrier; APOE, Apolipoprotein E; BACE, Beta secretase; PSEN, Presenilin; hiPSCs, Human induced pluripotent stem cells; RBV, Ribavirin; hESCs, Human embryonic stem cells; NSCs, Neural stem cells; EB, Embryoid body; VZ, Ventricular zone; CP, Cortical plate; SVZ, Subventricular zone; MAP2, Microtubule-associated protein 2; SOX2, SRY-box transcription factor 2; Iba-1, Ionized calcium binding adaptor molecule-1; IL, Interleukin; TNFα, Tumor necrosis factor α; P/S, Penicillin/Streptomycin; PBS, Phosphate buffer saline; DPBS, Dulbecco’s phosphate-buffered saline; GO, Gene ontology; KEGG, Kyoto Encyclopedia of Genes and Genomes; RNA-seq, RNA-sequencing; PFA, Paraformaldehyde; RT-PCR, Real-time polymerase chain reaction; ASCL1, Achaete-scute Family bHLH; Transcription Factor 1; TUJ1, Neuronal Class III β-Tubulin; CD11b, Recombinant Integrin Alpha M; GFAP, Glial fibrillary acidic protein; CD68, Cluster of differentiation 68; HLADR, Human leukocyte antigen—DR isotype; CX3CR1, CX3C chemokine receptor 1; GAPDH, Glyceraldehydes-3-phosphate; bFGF, Basic fibroblast growth factor; ROCK, Rho-associated, coiled-coil containing protein kinase; DMEM, Dulbecco’s Modified Eagle Medium; DAPI, 4′,6-diamidino-2-phenylindole, SATB2, Special AT-rich sequence-binding protein 2; CTIP2, Chicken ovalbumin upstream promoter transcription factor (COUP-TF)-interacting protein 2.
