# Supplementary material for: Cerebral Organoids for Modeling of HSV-1-Induced-Amyloid β Associated Neuropathology and Phenotypic Rescue
Source: Int J Mol Sci. 2022 May 26;23(11):5981. doi: 10.3390/ijms23115981 (PMC9181143; doi:10.3390/ijms23115981)
Supplement: Supplementary file 1 [file ijms-23-05981-s001.zip › ijms-1728714-supplementary.pdf]

Figure S1

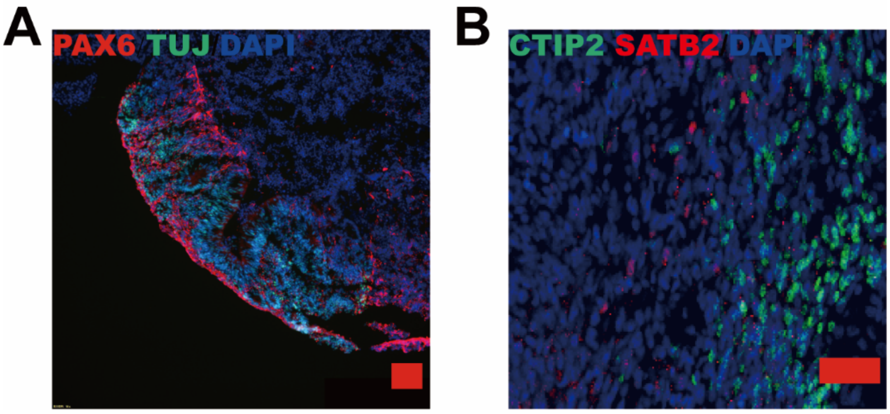

Figure S1. The cerebral layer of cerebral organoids at different stage. (**A**) The immunofluorescence staining for PAX6 (dorsal forebrain NSC marker) and TUJ (newborn neuron marker) in the cerebral organoids at the D45. (**B**) The immunofluorescence staining for SATB2 (late-born neuron marker) and CTIP2 (early-born neuron marker) in the cerebral organoids at D68. Scale bar 100 μm.
